# Aetiology of acute respiratory infection in Vientiane, Lao PDR, from a case–control study

**DOI:** 10.1038/s41598-026-41321-9

**Published:** 2026-03-01

**Authors:** John D. Hart, David A. B. Dance, Keoudomphone Vilivong, Toukta Bounkhoun, Souphatsone Phommachan, Ruth Lim, Jana Lai, Melinda Morpeth, Catherine Satzke, Mayfong Mayxay, Xavier de Lamballerie, Paul N. Newton, Fiona M. Russell, Audrey Dubot-Pérès

**Affiliations:** 1 Infection, Immunity and Global Health, Murdoch Children’s Research Institute, Melbourne, VIC Australia; 2 Department of Paediatrics, The University of Melbourne, Melbourne, VIC Australia; 3 Microbiology Laboratory, Lao-Oxford-Mahosot Hospital-Wellcome Trust Research Unit (LOMWRU), Mahosot Hospital, Vientiane, Lao PDR; 4 Centre for Tropical Medicine and Global Health, Nuffield Department of Clinical Medicine, University of Oxford, Oxford, UK; 5 Faculty of Infectious and Tropical Diseases, London School of Hygiene and Tropical Medicine, London, UK; 6 National Centre for Epidemiology & Population Health, Australian National University, Canberra, Australia; 7 The Royal Children’s Hospital, Melbourne, Australia; 8 Department of Microbiology and Immunology, The University of Melbourne at the Peter Doherty Institute for Infection and Immunity, Melbourne, VIC Australia; 9 Institute of Research and Education Development, University of Health Sciences, Vientiane, Lao PDR; 10 Unité Des Virus Émergents (UVE: Aix-Marseille Univ, Università Di Corsica, IRD 190, Inserm 1207, IRBA), Marseille, France; 11 Mahidol-Oxford Tropical Medicine Research Unit (MORU), Mahidol University Faculty of Tropical Medicine, Bangkok, Thailand

**Keywords:** Acute respiratory infection, Laos, Children, Respiratory syncytial virus, Case–control study, Diseases, Health care, Medical research, Microbiology, Risk factors

## Abstract

Acute respiratory infections (ARI) remain a major cause of child mortality in low- and middle-income countries. However, the risk factors for ARI are poorly understood in low-income settings, and ARI aetiology is changing, driven by vaccination. There are very limited data from Lao PDR (Laos) on ARI aetiology and risk factors to support health policy decisions. This study aimed to investigate the aetiology of hospitalised ARI, and describe risk factors associated with hospitalised ARI, in children under 5 years of age in Laos. We conducted a case–control study at Mahosot Hospital, Laos, enrolling children under five years of age hospitalised with ARI, and community controls matched on age, sex and time of recruitment. Demographics and clinical characteristics were collected, and throat swabs taken. Swabs were analysed using probe-based real-time polymerase chain reaction (PCR) to detect bacterial and viral microorganisms. Risk factors for ARI were determined through regression analysis, and microorganism-specific attributable fractions (AF) were calculated to estimate each microorganism’s contribution to hospitalised ARI. We enrolled 307 cases and 564 controls over 12 months in 2016/17. Microorganisms were detected in 93.8% of cases and 58.9% of controls. Respiratory syncytial virus (RSV) was the leading viral cause of hospitalised ARI, attributed to 29.6% of cases, followed by influenza viruses (11.6%). *H. influenzae* was attributed to 40.8% of cases. RSV exhibited clear seasonality, peaking during the wet season. Exclusive breastfeeding for 3 months (OR: 0.62; 95% CI 0.45–0.86), and being up to date with pneumococcal conjugate vaccination (odds ratio: 0.6; 95% CI 0.41–0.80), were associated with a lower risk of hospitalised ARI; while low birth weight (OR: 2.91; 95% CI 1.63–5.28), and household smoking (OR: 3.07; 95% CI 2.25–4.18), were associated with increased risk. RSV and *H. influenzae* remain major causes of ARI in Laos. The findings highlight the potential benefit of tailoring interventions to the local context, including vaccination and risk mitigation strategies, to reduce the burden of ARI in Laos and other low and middle-income countries.

**Supplementary Information:**

The online version contains supplementary material available at 10.1038/s41598-026-41321-9.

## Introduction

Acute respiratory infection (ARI) is the leading cause of child mortality globally, claiming the lives of more than 500,000 children under five years of age annually^1^. The greatest burden of ARI falls in the least developed settings, with 99% of global ARI deaths occurring in low and middle-income countries^2^. The aetiological landscape of ARI is changing globally, associated with factors including the use of pneumococcal conjugate vaccine (PCV), *Haemophilus influenzae* type b vaccine and the recent licensure in some settings of respiratory syncytial virus (RSV) vaccines and monoclonal antibodies. In addition, with rapid urbanisation and changes in socioeconomic status in low and middle-income countries, risk factors for ARI are continually changing^3^. The Pneumonia Etiology Research for Child Health (PERCH) study enrolled 4232 children hospitalised with severe pneumonia and matched controls in seven countries in Africa and Asia, and found that viruses were the primary cause, accounting for 61% of cases, with RSV responsible for 31% of all cases^4^. Leading risk factors for ARI globally include low birth weight, non-exclusive breastfeeding, malnutrition, indoor air pollution, household crowding, and under-vaccination, but these factors vary considerably by country, highlighting the importance of local data^5^.

Global efforts are currently underway to better understand the aetiological causes of ARI and child mortality to guide health policy and vaccine development research^6^. The Child Health and Mortality Prevention Surveillance study, using minimally invasive autopsy, identified lower respiratory infection among the leading immediate and underlying causes of death among infants and children in low-income settings, alongside malnutrition, malaria and sepsis. However, determining the aetiology of ARI is particularly challenging since sampling the lower respiratory tract is difficult and rarely conducted for routine diagnosis, and the sensitivity of blood culture for pneumonia aetiological diagnosis is relatively low^7^. Detection from the upper respiratory tract is possible and, with advances in molecular diagnostics, it is highly sensitive and can identify a broad range of bacterial and viral microorganisms. Nonetheless, identification of some organisms may represent asymptomatic carriage, not necessarily the cause of lower respiratory infections. Furthermore, high quality surveillance is not often implemented in low- and middle-income settings despite local data being critical for identifying the leading causes and risk factors in each setting, to tailor policy to countries’ specific requirements. One approach to improve the determination of aetiological causes of ARI involves enrolling controls from the same community to compare upper respiratory microorganism detection between controls and cases and enable estimation of the proportion of ARI cases attributed to each microorganism.

Lao PDR (Laos) is a tropical country in South East Asia, characterized by distinct wet and dry seasons, that has high under five child mortality (43 per 1,000 live births in 2021, World Bank Data). There are relatively limited data from Laos on the characteristics and aetiology of ARI. Data from hospitalised patients in northeastern Laos in 2019–2020 showed that more than half of cases were attributable to viruses, mainly influenza A virus, influenza B virus, human metapneumovirus (HMPV), and RSV^8^. Here, we present a case–control study to investigate the aetiology and describe individual factors associated with hospitalised ARI in children under 5 years of age at Mahosot Hospital in Vientiane, the capital of Laos.

## Materials and methods

### Study site

This prospective case–control study was conducted from 27 June 2016 to 27 June 2017 at Mahosot Hospital, Vientiane, Laos, a tertiary hospital with 400 beds that admits approximately 2,000 patients per month.

### Case recruitment

Children under 5 years of age, admitted to a paediatric ward (general paediatric, paediatric infectious diseases, or paediatric intensve care unit) were eligible for enrolment if they presented with ARI: < 14 day history of symptoms; fever (axillary temperature > 38.0 °C) or history of fever and at least one respiratory symptom (dyspnoea, cough, rhinitis); or abnormal pulmonary auscultation on physical examination. Study physicians collected sociodemographic and medical history from the child’s birth record book and PCV vaccination record, using a questionnaire with study families, physical examination and medical records. For all participants, we collected age, sex, ethnicity, clinical history (including birth history and underlying comorbidities), PCV vaccination history, and household/environmental exposures and other potential ARI risk factors, selected a priori based on biological plausibility and prior evidence as determinants of ARI risk in young children^9^. Regarding ethnicity, Lao Loum (literally “lowland Lao”) refers to Lao-Tai groups that mainly inhabit the Mekong lowlands. Lao Loum comprise approximately 92% of the population of Vientiane Capital, and 62% of the population of Laos overall^10^. Non-Lao Loum groups are more likely to reside in highland areas and generally experience greater geographic and ethno-linguistic barriers to health care^10^.

### Control recruitment

Children attending Mahosot Hospital for routine immunisations were recruited as controls. Two controls were recruited per case. Inclusion criteria for controls were: no respiratory signs or symptoms and no fever within the last 14 days; normal respiratory rate (< 60/minute for infants aged < 2 months, < 50/minute for infant aged 2 months to < 1 year, < 40/minute for children aged 1 year or older). Controls were matched on age (± 3 months) with cases and were recruited within one month of case enrolment, because of the variation in ARI risk and respiratory pathogen detection by age among young children.

### Sample collection and laboratory assays

Throat swab specimens were collected from all cases and controls. Swabs were placed in 1 mL viral transport medium (Sigma Virocult®, MWE). Virocult vials were transported to the laboratory within 2 h in a cool box. Swabs were squeezed, and the media aliquoted and stored at − 80 °C until the laboratory assays were performed. For all cases and controls, microorganism nucleic acids were extracted from 100 µL of throat swab sample using the Qiagen Cador Pathogen 96 QIAcube HT kit (for samples collected in 2016), or Qiagen EZ1 virus mini kit (for samples collected in 2017) following the manufacturer’s instructions, with an elution of 90 µL. Extracts were tested using previously published singleplex reverse-transcription probe based real-time polymerase chain reaction (qRT-PCR) assays targeting 7 respiratory microorganisms (the leading causes of ARI): influenza A virus^11^, influenza B virus^12^, RSV A/B^13^, human rhinovirus (HRV) (in house; forward 300 nM: 5′WGCCVGCGTGGCKGCC 3′, forward 300 nM: 5′AGCCYGCGTGGTGCCC 3′, reverse 300 nM: 5′GAAACACGGACACCCAA AGTAGT 3′, probe 133 nM: 5′FAM-CTCCGGCCCCTGAATGYGGCTAA-TAMRA 3′), enterovirus (EV)^14^, *H. influenzae*^15^, and a two-plex containing internal control and HMPV A/B^16^. For each system, primers and probe mix were lyophilised, as previously described^17^. Testing was performed using EXPRESS One-Step Superscript™ qRT-PCR Kit (Thermofisher, for samples collected in 2016), or using GoTaq® Probe 1-Step RT-qPCR System (Promega, samples collected in 2017) from 5 µL of nucleic acids, in a final volume of 20 µL.

All amplification and detection was performed with the CFX Real-time PCR system instrument (Bio-Rad). Positive and negative (no template) controls were included in each PCR run. The q(RT-)PCR assays were considered positive if the (quantification cycle) Cq value was < 35. As there is no recommended Cq cut-off for positivity for the assays conducted, additional analyses were performed using Cq cutoff < 40.

### Statistical analysis

No formal a priori sample size calculation was performed as enrolment was determined by eligible presentations during the study period. All analyses were conducted using Stata version 18. Participant data were summarized as number and percentage for cases and controls. Logistic regression models were used to calculate odds ratios (ORs) with 95% confidence intervals (CIs), showing the unadjusted strength and direction of association between each clinical and demographic characteristic and the outcome (hospitalisation with ARI). Given our aim to present a range of potential risk factors rather than to adjust for confounding of a specific exposure-outcome relationship, multivariable analysis was not conducted.

The microorganisms identified, with Cq cutoff < 35, were compared between cases and controls using logistic regression and presented as ORs with 95% CIs. The ORs were used to calculate the attributable fraction among the exposed (AFE; the proportion of cases found to be positive for a given microorganism, for whom the disease can be attributed to that microorganism) as: 1 – (1/OR). The attributable fraction (AF; the proportion of cases for whom the disease can be attributed to a given microorganism) for each microorganism was calculated as prevalence of the microorganism in cases multiplied by the AFE^18^. Similar analyses were conducted using Cq cutoff < 40, and by season (wet: May to October and dry: November to April).

Box plots of PCR Cq values for each microorganism were performed to visualise the values in cases and controls.

### Ethics

We obtained written informed consent from legal guardians of participants before recruitment to the study. The study was conducted according to the protocol approved by the National Ethics Committee for Health Research, Ministry of Health (Vientiane, Laos; NECHR Ref. 057/2013); the Oxford University Tropical Ethics Research Committee (Oxford, UK; OxTREC Ref. 1050-13); and The Royal Children’s Hospital Human Research Ethics Committee (Melbourne, Australia; HREC Ref. 33177 A). All study procedures were performed in accordance with relevant guidelines and regulations.

## Results

**Fig. 1:**
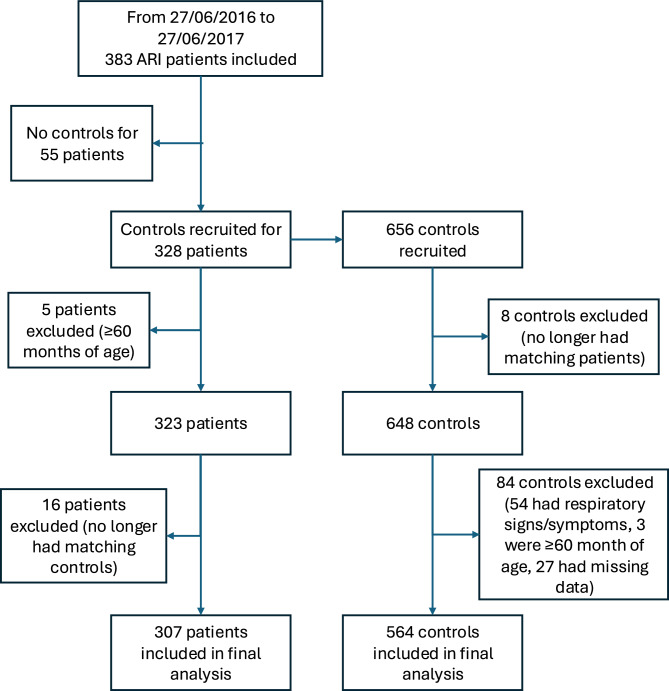
Flow chart of case and control recruitment.

This study enrolled 307 cases and 564 controls (Fig. 1). A protocol deviation occurred in 100 pairs with a difference in age between cases and controls exceeding three months. However, there was no difference in age distribution between cases (median 14.3 months, interquartile range 7.2–25.5) and controls (median 14.7 months, interquartile range 6.6–25.0) (Wilcoxon rank-sum test, *P* = 0.57) and the pairs with discrepant ages are included in the analysis.

### Factors associated with hospitalised ARI

Table 1 summarises participant demographic and clinical characteristics, and ORs for potential risk factors for ARI requiring hospitalisation between cases and controls. There were greater odds of being a case for those with non-Lao Loum ethnicity (OR: 3.41, 95% CI 1.85–6.29); having a smoker in the house (OR: 3.07, 2.28–4.14), low birth weight (OR: 2.91, 1.68–5.04), unimproved drinking water source (OR: 6.54, 4.24–10.07) and being underweight (OR: 2.47, 1.61–3.78). Detection of any respiratory microorganism was associated with greatly increased odds of being a case (OR: 24.1, 16.7–34.8). Exclusive breast feeding for 3 months (OR: 0.59, 0.43–0.81), and being up to date with pneumococcal conjugate vaccine (OR: 0.80, 0.59–1.08), were associated with lower odds of ARI requiring hospitalisation, versus controls.

**Table 1:** Characteristics of cases and controls.

	Total, N = 871, n (%)	Cases, N=307, n (%)	Controls, N=564, n (%)	Crude odds ratio (95% CI)
Demographic and household characteristics				
Age				
< 6 months	204 (23.7)	68 (22.2)	136 (24.5)	–
6–11 months	170 (19.7)	62 (20.3)	108 (19.4)	1.15 (0.75–1.76)
12–23 months	245 (28.4)	87 (28.4)	158 (28.4)	1.10 (0.74 (1.63)
24–59 months	243 (28.2)	89 (29.1)	154 (27.7)	1.16 (0.78–1.71)
Gender				
Male	462 (54.5)	177 (57.7)	285 (52.8)	–
Female	385 (45.5)	130 (42.4)	255 (47.2)	0.82 (0.62–1.09)
Ethnicity*				
Lao Loum	812 (94.5)	277 (90.2)	535 (96.9)	–
Other	47 (5.5)	30 (9.8)	17 (3.1)	3.41 (1.85–6.29)
Exposed to indoor air pollution**	543 (62.9)	183 (59.6)	360 (64.7)	0.80 (0.60–1.07)
Low mother’s education				
University education	274 (32.0)	83 (27.8)	191 (34.4)	–
No university education	581 (68.0)	216 (72.2)	365 (65.6)	1.36 (0.99–1.88)
Smoker in house	280 (32.7)	150 (48.9)	130 (23.7)	3.07 (2.28–4.14)
Family income below the LMIC poverty line^†^	304 (34.9)	108 (35.2)	196 (34.8)	1.02 (0.76–1.36)
Kindergarten attendance	661 (78.2)	234 (76.2)	427 (79.4)	1.20 (0.86–1.68)
Unimproved water source^‡^	115 (14.3)	80 (30.9)	35 (6.4)	6.54 (4.24–10.07)
Not flush or ventilated pit latrine	9 (1.1)	3 (1.0)	6 (1.1)	1.13 (0.28–4.53)
Clinical characteristics				
Low birth weight (< 2,500 g)	57 (6.9)	34 (11.5)	23 (4.3)	2.91 (1.68–5.04)
Exclusive breast feeding for 3 months	631 (73.8)	206 (67.0)	425 (76.6)	0.59 (0.43–0.81)
Yes				
Premature (< 37 weeks gestation)	99 (11.9)	4 (1.3)	95 (18.1)	0.06 (0.02–0.16)
Underweight^§^	100 (12.8)	58 (19.2)	42 (8.8)	2.47 (1.61–3.78)
Delivery				
Vaginal	727 (85.5)	259 (84.4)	468 (86.2)	-
Caesarean	123 (14.5)	48 (15.6)	75 (13.8)	1.16 (0.78–1.71)
Up to date PCV^‖^	510 (62.2)	159 (58.7)	351 (63.9)	0.80 (0.59–1.08)
Detection of any microorganism^¶^	296 (34.1)	232 (75.6)	64 (11.4)	24.1 (16.7–34.8)

* Lao Loum is the ethnic majority Lowland Lao, while “non-Lao Loum” refers to other ethnic groups in Lao PDR.

**Exposed to indoor air pollution if cooking place is inside the house and cooking fuel is wood or coal.

^†^Lower Middle Income Class Poverty Line in 2018: ≤ $2.15 per person per day^29^.

^‡^Unimproved water source if not piped drinking water in residence, public faucet or protected well.

^§^Weight-for-age ≤ -2 standard deviations (SD) of the WHO Child growth standards median.

^‖^Up to date defined as adequate number of PCV doses ≥ 14 days before enrolment: for children < 12 months of age, two or more PCV doses; for children ≥ 12 months, two doses in the first year of life or at least 1 dose after the age of 12 months.

^¶^Detection with PCR Cq < 35 of any of influenza viruses, RSV, HRV, EV, HMPV, or *H. influenzae*.

Data missing for age: n = 9 participants, gender n = 24, ethnicity n = 12, indoor air pollution n = 8, mother’s education n = 16, smoker in house n = 16, kindergarten attendance n = 26, water source n = 65, sanitation n = 18, birth weight n = 39, breast feeding n = 16, prematurity n = 40, weight n = 91, hot bed n = 670, delivery n = 21, PCV vaccination status n = 51, microorganism detection n = 2.

### Microorganisms detected

At least one microorganism was detected in 232/307 (75.6%) cases and 64/562 (11.4%) controls. Two or more microorganisms (up to three in each participant) were detected in 83/307 (27.0%) cases and 7/562 (1.2%) controls.

All microorganisms detected (influenza A virus, influenza B virus, RSV, HRV, EV, HMPV and *H. influenzae*) were much more likely to be identified in cases than controls (ORs between 6.5 and 79.7) (Table 2). Influenza A virus and HMPV were not detected in controls. After Influenza A virus and HMPV, RSV, which was attributed to 29.6% of cases, was the microorganism most likely to be the cause of disease when detected in the throat (AFE 98.7, 95% CI 96.1–99.7).

**Table 2:** Microorganisms detected by PCR from throat swabs in hospitalised children with acute respiratory infection and matched healthy controls, July 2016-July 2017.

Microorganism	Cases N = 307 n (%)	Controls N = 562 n (%)	Odds ratio (95%CI)	*P*-value*	Attributable fraction in the exposed^#^, % (95%CI)	Attributable fraction^>^, %
Viruses						
Influenza A	23 (7.5)	0 (0.0)	–	–	–	–
Influenza B	14 (4.6)	3 (0.5)	8.9 (2.5–48.6)	0.001	88.8 (60.0–97.9)	4.1 (2.8–4.5)
Influenza A or B	37 (12.1)	3 (0.5)	25.6 (8.0–130.7)	< 0.001	96.1 (87.5–99.2)	11.6 (10.6–12.0)
RSV	92 (30.0)	3 (0.5)	79.7 (25.9–396.2)	< 0.001	98.7 (96.1–99.7)	29.6 (28.8–29.9)
HRV	17 (5.4)	5 (0.9)	6.5 (2.3–22.8)	< 0.001	84.6 (56.5–95.6)	4.6 (3.1–5.2)
EV	13 (4.2)	2 (0.4)	12.4 (2.8–113.4)	0.001	91.9 (64.3–99.1)	3.9 (2.7–4.2)
HMPV	14 (4.6)	0 (0.0)	–	–	–	–
Bacteria						
*H. influenzae*	144 (46.9)	58 (10.3)	7.7 (5.3–11.1)	< 0.001	87.0 (81.1–91.0)	40.8 (38.0–42.7)

Cq cutoff < 35 used for positivity.

*P-value from logistic regression.

^**#**^AFE: the proportion of cases found to be positive for a given microorganism, for whom the disease can be attributed to that microorganism, calculated as: 1 – (1/OR).

^>^AF: the proportion of cases for whom the disease can be attributed to a given microorganism, calculated as prevalence in cases multiplied by the attributable fraction in the exposed^18^.

**Fig. 2:**
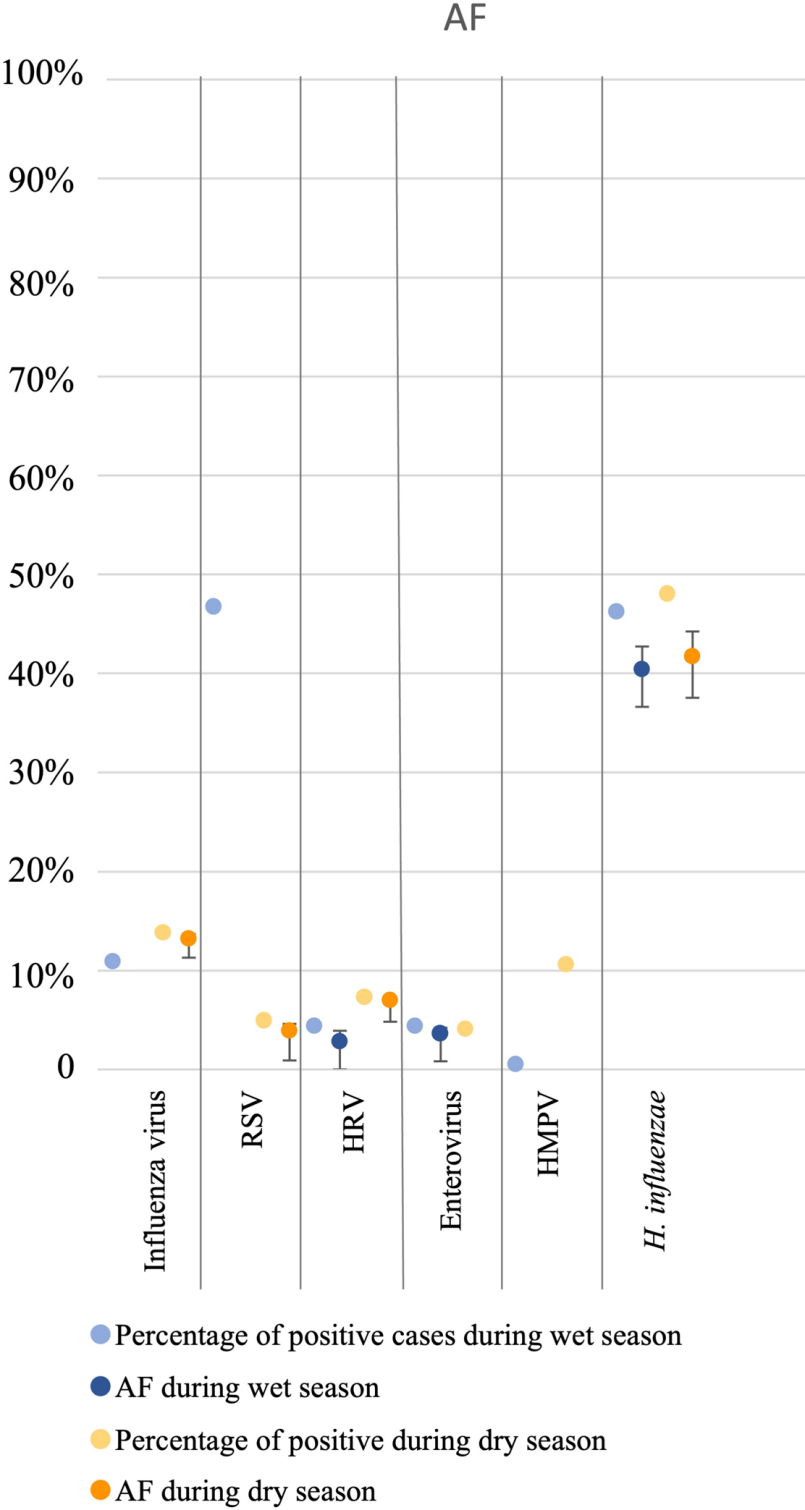
Comparison of attributable fractions during wet and dry season.Wet season: May to October, dry season: November to April. AF: the attributable fraction is the proportion of cases for whom the disease can be attributed to the microorganism detected, calculated as prevalence of the microorganism detection in cases multiplied by the attributable fraction among the exposed (AFE). AFE: probability for the microorganism detected to be the cause of the disease, calculated as: 1-(1/OR). OR: odds ratio calculated from percentage of cases and percentage of controls positive for the given microorganism. For influenza virus and RSV during wet season and enterovirus during dry season, AF could not be calculated (so is not displayed) since these viruses were not detected in any controls.

Seasonality of microorganism detection is shown in Table 3. RSV was the most notably seasonal microorganism (Fig. 2, Table 3), identified in 86/184 (46.7%) cases and 0/257 (0%) controls during the wet season, and only 6/123 (4.9%) cases and 3/298 (1.0%) controls during the dry season.

**Table 3:** Microorganisms detected by PCR during the wet and dry seasons from throat swabs in hospitalised children with acute respiratory infection and matched healthy controls, July 2016-July 2017.

	Wet season						Dry season					
Microorganism	Cases N = 184 n (%)	Controls N = 257 n (%)	Odds ratio (95%CI)	*P*-value*	AFE^#^, % (95%CI)	AF^>^, %	Cases N = 123 n (%)	Controls N = 298 n (%)	Odds ratio (95%CI)	*P*-value*	AFE^#^, % (95%CI)	AF^>^, %
Viruses												
Influenza A	12 (6.5)	0 (0.0)	–	–	–	–	11 (8.9)	0 (0.0)	–	–	–	–
Influenza B	8 (4.4)	0 (0.0)	–	–	–	–	6 (4.9)	2 (0.7)	7.6 (1.5–38.1)	0.014	86.8 (33.8–97.4)	4.2 (1.6–4.8)
Influenza A or B	20 (10.9)	0 (0.0)	–	–	–	–	17 (13.8)	2 (0.7)	23.7 (5.4–104.5)	< 0.001	95.8 (81.5–99.0)	13.2 (11.3–13.7)
RSV	86 (46.7)	0 (0.0)	–	–	–	–	6 (4.9)	3 (1.0)	5.0 (1.2–20.5)	0.024	80.2 (19.4–95.1)	3.9 (0.9–4.6)
HRV	8 (4.4)	4 (1.6)	2.9 (0.9–9.7)	0.089	65.2 (-17.3–89.7)	2.8 (-0.8–3.9	9 (7.3)	1 (0.3)	23.4 (2.9–187.2)	0.003	95.7 (66.0–99.5)	7.0 (4.8–7.3)
EV	8 (4.4)	2 (0.8)	5.8 (1.2–27.6)	0.027	82.7 (17.8–96.4)	3.6 (0.8–4.2)	5 (4.1)	0 (0.0)	–	–	–	–
HMPV	1 (0.5)	0 (0.0)	–	–	–	-	13 (10.6)	0 (0.0)	–	–	–	–
Bacteria												
*H. influenzae*	85 (46.2)	25 (9.7)	8.0 (4.8–13.2)	< 0.001	87.4 (79.2–92.4	40.4 (36.6–42.7)	59 (48.0)	32 (10.7)	7.7 (4.6–12.8)	< 0.001	87.0 (78.3–92.2)	41.7 (37.5–44.2)

Seven participants had missing data for season.

*P-value from logistic regression.

^**#**^AFE: the proportion of cases found to be positive for a given microorganism, for whom the disease can be attributed to that microorganism, calculated as: 1 – (1/OR).

AF: the proportion of cases for whom the disease can be attributed to a given microorganism, calculated as prevalence in cases multiplied by the attributable fraction in the exposed^18^.

Wet season: May to October.

Cq cutoff < 35 used for positivity.

**Fig. 3:**
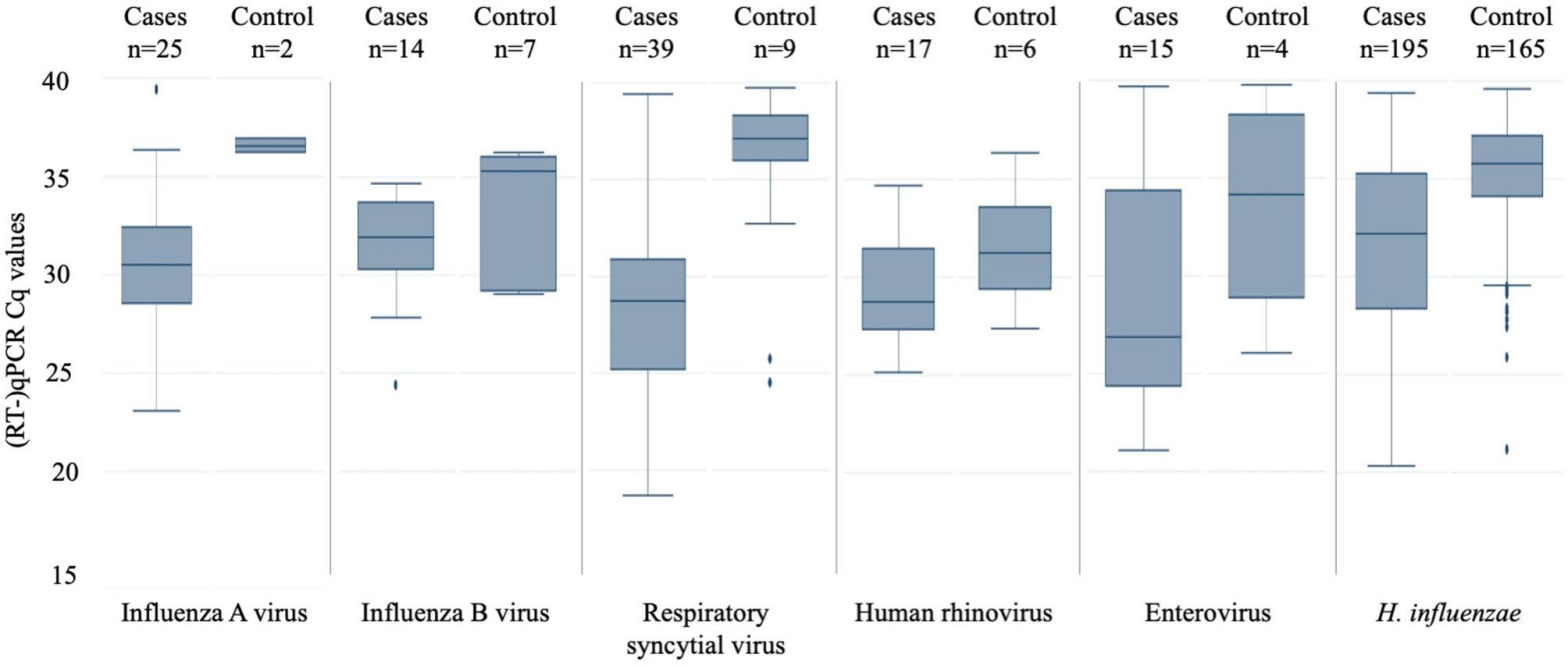
Comparison of Cq value obtained by PCR for the different microorganisms tested in throat swabs from cases and controls. Horizontal line indicates median Cq value. Boxes are indicating 25th and 75th percentile interquartile range and vertical lines maximum and minimum Cq values.

**Fig. 4:**
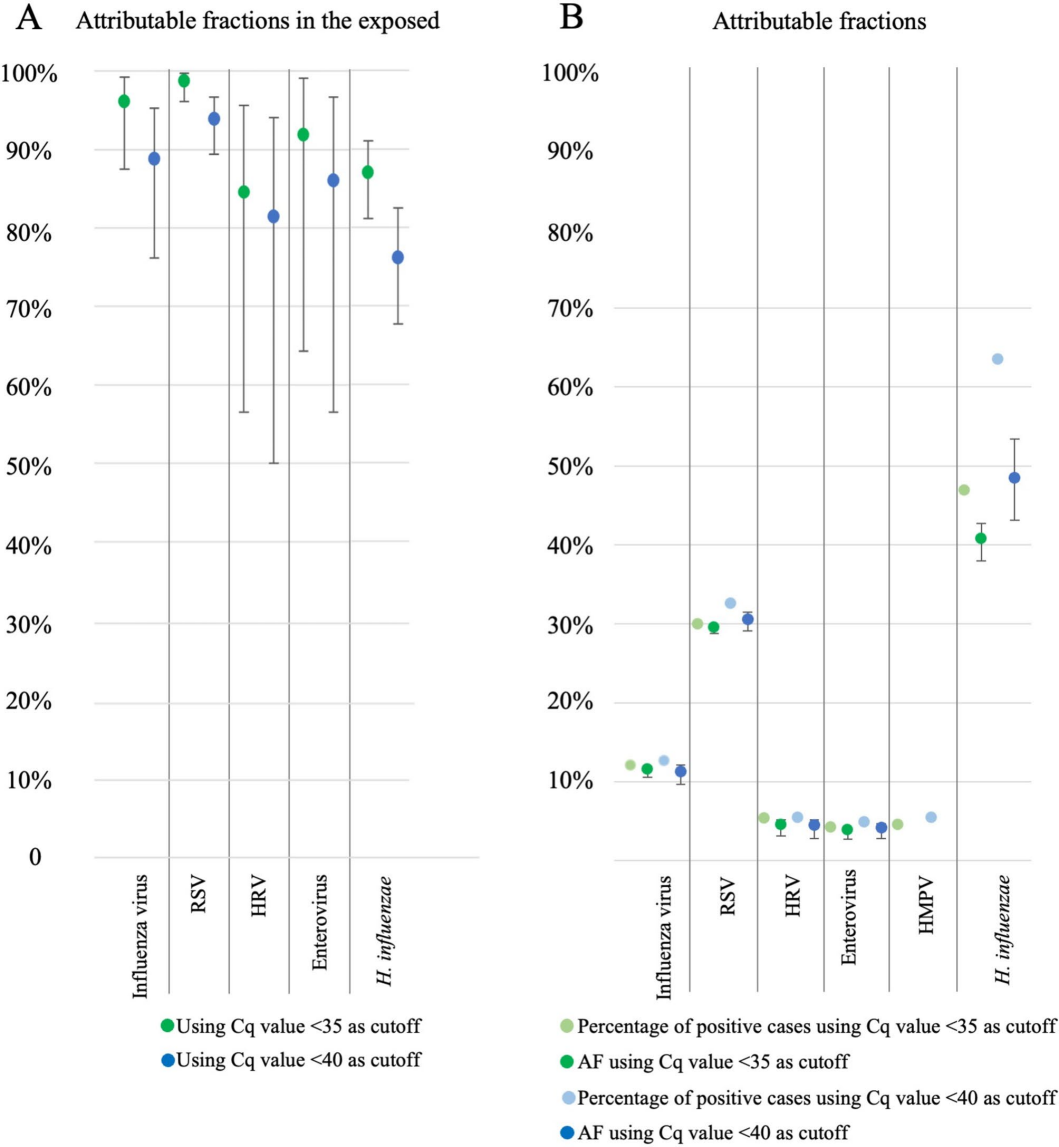
Comparison of attributable fractions when cutoff for microorganism detection by PCR is set as Cq < 35 or Cq < 40. A: Attributable fraction among the exposed (AFE) correspond to the probability for the microorganism detected to be the cause of the disease, calculated as: 1-(1/OR). OR: odds ratio calculated from percentage of cases and percentation of controls found positive for the given microorganism. B: AF: Attributable fraction (AF) is the proportion of cases for whom the disease can be attributed to the microorganism detected, calculated as prevalence of the microorganism detection in cases multiplied by the attributable fraction among the exposed (AFE).

PCR Cq values were lower in cases compared to controls (Fig. 3). The data were skewed towards higher Cq values in controls but not in cases for most microorganisms (Supplementary Fig. 1). The attributable fractions among the exposed (AFE) (the probability the detected microorganism is the cause of the disease) were higher for all microorganisms using Cq < 35 compared to Cq < 40 (Fig. 4, Tables 2 and 3, Supplementary Tables 1 and 2). However, the attributable fractions (the proportion of cases for whom the disease can be attributed to a given microorganism), were similar using cutoffs Cq < 35 and Cq < 40, except for *H. influenzae* for which it was substantially lower (40.8% versus 48.5%, p = 0.06) (Fig. 4, Tables 2 and 3, Supplementary Tables 1 and 2).

## Discussion

Our study showed that RSV was the leading viral cause of ARI requiring hospitalisation, as the aetiological agent in 29.6% of ARI cases, followed by influenza viruses in 11.6%. HRV, EV and HMPV were less frequent, each detected in around 5% of cases. All the viruses were rarely detected among controls. Therefore, the attributable fractions among the exposed, corresponding to the probability of the microorganism detected being the cause of disease, were high for all viruses (> 80%). For clinical practice, detection of those viruses in the upper respiratory tract is a reliable diagnostic, and facilitates simple estimation of the proportion of cases caused by each virus. This is in contrast to *H. influenzae* detection rates, which overestimate its aetiological role: *H. influenzae* was detected in 46.9% of cases but was also relatively frequently detected in controls, 10.3%. This reflects more common asymptomatic colonisation with bacterial compared to viral microorganisms^19^.

In the current study, we identified the presence of a smoker in the household, low birth weight, being underweight, and the use of an unimproved water source for drinking, as factors associated with hospitalisation with acute respiratory infections (ARI). Modifiable risk factors, particularly those related to nutritional status and lower quality living environments are generally recognised to increase the risk of ARI and adverse outcomes^5,20,21^. Exclusive breast feeding for 3 months and being up to date with PCV vaccination were associated with a lower risk of hospitalisation with ARI. These factors could be markers of socioeconomic status and health seeking behaviour. However, the results support our previous study in the same setting showing that PCV vaccination protects against hypoxic pneumonia^22^. Surprisingly, our study identified premature birth to be associated with lower odds of being hospitalised with ARI. This is likely explained by over-representation of premature children at the hospital vaccination clinic where recruitment took place, as premature children are more often delivered at specialised hospitals and monitored for vaccination at those hospital vaccination clinics.

We found that RSV cases were highly seasonal. Understanding RSV seasonality, which is predictable in temperate regions, but often less defined in the tropics, will be important for Laos and other countries in the region as interventions to prevent RSV disease are introduced^23–25^. In our study, we summarized seasonality using Laos’s wet and dry seasons rather than month-by-month estimates, given our relatively small numbers of participants. Research is ongoing in other settings to define RSV seasonality, and how it may vary even within countries, to help define the optimal timing of RSV immunisation programmes – in China, for example, seasonal patterns have been shown to vary considerably across the country^25^. An approach similar to many high income settings, with seasonal implementation of monoclonal antibody injections and maternal vaccination, would be appropriate in this seasonal setting, although low and middle income countries may use policies focused on maternal vaccination given the current high cost of monoclonal antibodies^26^. Seasonal ARI management strategies may include influenza vaccine, as determined by the local epidemiology, particularly among higher risk infants and children.

The comparison of microorganism detection between cases and controls permitted a more accurate estimation of the proportion of hospitalised children with ARI due to that microorganism. The attributable fraction for *H. influenzae*, although lower than the detection rate, remains high (40.8%). Our previous work identified *H. influenzae* carriage among 44–50% of healthy infants in Fiji, and 28% of 12–24-month-olds in Indonesia (compared to 64% of cases and 29% of controls in our study)^27^. *Haemophilus influenzae* type b vaccination was introduced in Laos in 2009, with more than 99% of children under five years of age subsequently showing at least short term serological protection against *H. influenzae*^28^*.* However, our study findings show that *H. influenzae* remains an important aetiological cause of pneumonia in this setting.

The case control approach enables a better estimation of the aetiological role of microorganism detection in the upper respiratory tract in ARI. However, in clinical settings, only data from cases are available. Our analysis of Cq values in cases and controls shows lower microorganism loads in controls than in cases, likely representing asymptomatic carriage, or late resolved infections, and perhaps very minor infections. The generally left skewed histograms of Cq values for controls probably support the use of a relatively lower Cq cutoff for positivity, such as Cq < 35 that we selected. Interestingly, the *H. influenzae* attributable fraction was substantially higher when Cq cutoff < 40 was used (48.5%) compared to < 35 (40.8%), in contrast to the similar attributable fraction for other microorganisms. The lower Cq cutoff probably reduced an overestimate of the aetiological role of *H. influenzae* detected in the upper respiratory tract.

Our study has several limitations. First, while a similar proportion of our study sample (94.5%) were of Lao Loum ethnicity than in Vientiane overall (approximately 92%)^10^, a slightly greater proportion were Lao Loum among controls (96.9%) than cases (90.2%). This may be related to our recruitment of controls during routine immunisation, which could feasibly introduce bias. Our control selection may also tend to overrepresent children born prematurely and those with chronic diseases, as these children are more likely to be brought to the hospital clinics than to their local clinics. Accordingly, while the controls provide appropriate internal comparison within the same population, microorganism prevalence among controls may not be fully generalisable to the wider community. Second, the *H. influenzae* primers in our assay were not specific for *H. influenzae* type b, and in our study setting with *H. influenzae* type b vaccine use, may have detected other strains and non-typeable *H. influenzae*. Subtyping to establish *H. influenzae* subtypes would be a useful addition in future studies. Third, our PCR testing targeted a limited panel of pathogens and did not include parainfluenza, adenoviruses and seasonal coronaviruses, which likely resulted in under-ascertainment of viral detection. Finally, throat swabs were preferred in this study for ethical reasons, particularly for controls, as they cause less discomfort than nasopharyngeal swabs. However, not using nasopharyngeal swabs, which are the recommended sample for detection of some microorganisms, notably *S. pneumoniae,* limited the scope of microorganisms included in our study.

Our study shows that RSV followed by influenza are the dominant viral causes of ARI requiring hospitalisation in Laos, with RSV showing strong seasonality. *H. influenzae* also remains an important aetiological cause of hospitalised ARI in this setting. Viral detection in the upper respiratory tract strongly indicated causation, in contrast to *H. influenzae*, which was more commonly carried asymptomatically. We identified modifiable risk factors for ARI requiring hospitalisation, including nutritional status, household smoke exposure, and water quality, and showed that exclusive breastfeeding and PCV vaccination are associated with lower risk of hospitalised ARI. Our findings highlight the potential for integrated prevention strategies—particularly RSV and influenza immunisation programmes tailored to local seasonality, and addressing social risk factors for ARI requiring hospitalisation—to improve child health in Laos and the region.

## Supplementary Information

Supplementary Information.

## Data Availability

The individual participant data generated in this study are not publicly available due to data governance requirements. Specifically, data access is subject to approval by the Murdoch Children’s Research Institute (MCRI) Change Advisory Board, the Royal Children’s Hospital Human Research Ethics Committee, the National Ethics Committee for Health Research, Ministry of Health, Laos and the Oxford Tropical Research Ethics Committee. Academic researchers may request access to de-identified data. Requests will be reviewed by the MCRI Change Advisory Board and relevant ethics committees. If approved, data will be shared under a data sharing agreement.
