# Supplementary material for: Aetiology of acute respiratory infection in Vientiane, Lao PDR, from a case–control study
Source: Sci Rep. 2026 Mar 1;16:11492. doi: 10.1038/s41598-026-41321-9 (PMC13057353; doi:10.1038/s41598-026-41321-9)
Supplement: Supplementary file 1 — Supplementary Information. [file 41598_2026_41321_MOESM1_ESM.docx]

**Supplementary figure 1:** Histogram of PCR Cq values in cases and controls for each microorganism.


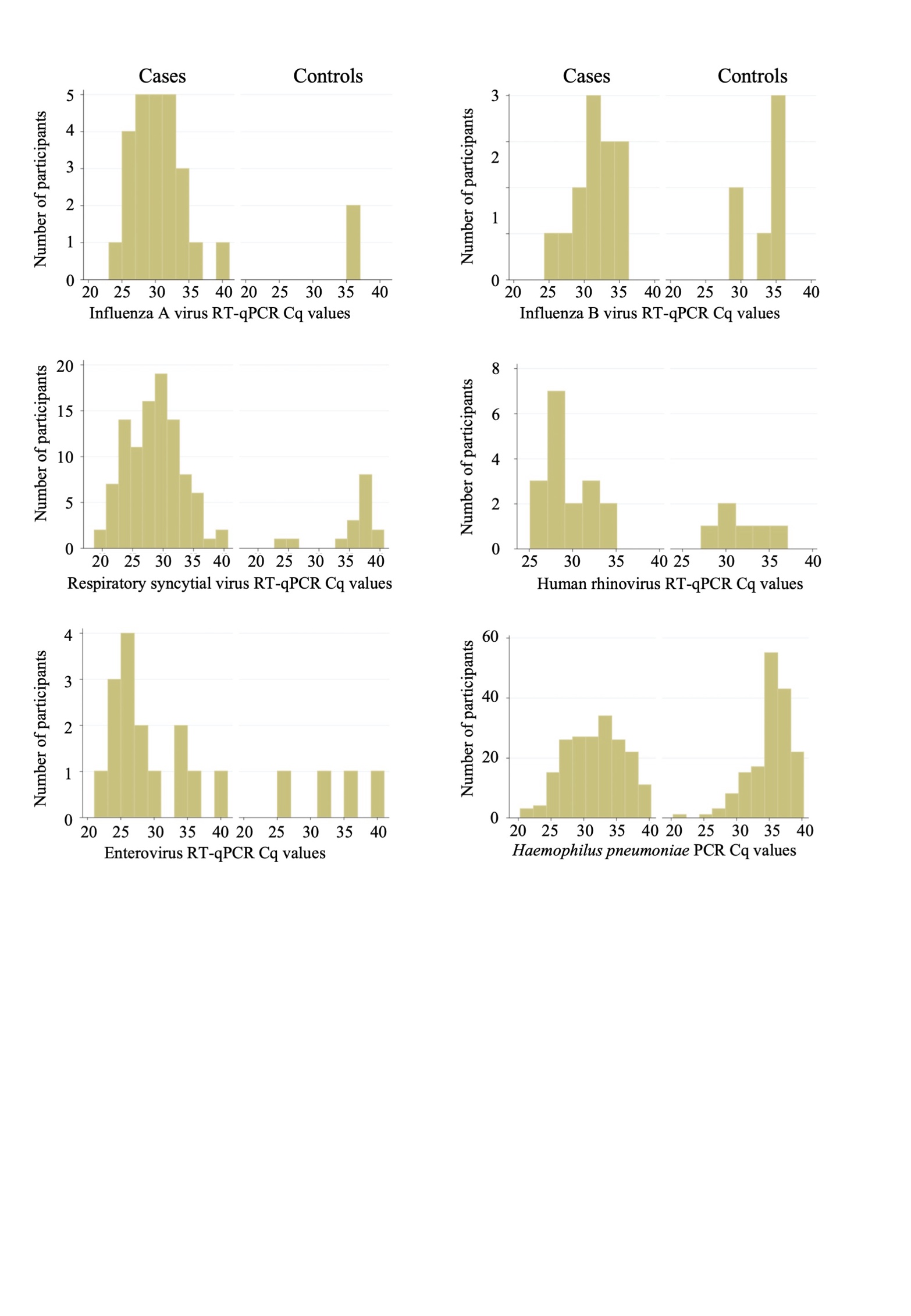


**Supplementary table 1:** Microorganisms detected by PCR, using cutoff for detection Cq<40, from throat swabs in hospitalised children with ARI and matched healthy controls, July-December 2016

| **Microorganism** | **Cases**  **N=307**  **n (%)** | **Controls**  **N=562**  **n (%)** | **Odds ratio (95%CI)** | ***P*-value*** | **Attributable fraction in the exposed^#^,**  **% (95%CI)** | **Attributable fraction^>^, %** |
| --- | --- | --- | --- | --- | --- | --- |
| Viruses |  |  |  |  |  |  |
| Influenza A | 25 (8.1) | 2 (0.4) | 24.8 (6.1-217.0) | <0.001 | 96.0 (83.6-99.5) | 7.8 (6.8-8.1) |
| Influenza B | 14 (4.6) | 7 (1.3) | 3.8 (1.4-11.2) | 0.003 | 73.7 (28.6-91.1) | 3.4 (1.3-4.2) |
| Influenza A or B | 39 (12.7) | 9 (1.6) | 8.9 (4.2-21.3) | <0.001 | 88.8 (76.2-95.3) | 11.3 (9.7-12.1) |
| RSV | 100 (32.6) | 16 (2.9) | 16.5 (9.4-30.6) | <0.001 | 93.9 (89.4-96.7) | 30.6 (29.1-31.5) |
| HRV | 17 (5.5) | 6 (1.1) | 5.4 (2.0-17.0) | <0.001 | 81.5 (50.0-94.1) | 4.5 (2.8-5.2) |
| EV | 15 (4.9) | 4 (0.7) | 7.2 (2.3-29.9) | <0.001 | 86.1 (56.5-96.7) | 4.2 (2.8-4.7) |
| HMPV | 17 (5.5) | 0 (0.0) | - | - | - | - |
| Bacteria |  |  |  |  |  |  |
| *H. influenzae* | 195 (63.5) | 165 (29.4) | 4.2 (3.1-5.7) | <0.001 | 76.2 (67.7-82.5) | 48.5 (43.1-53.4) |

**P*-value from logistic regression

**^#^**AFE: the proportion of cases found to be positive for a given microorganism, for whom the disease can be attributed to that microorganism, calculated as: 1 – (1/OR)

^>^AF: the proportion of cases for whom the disease can be attributed to a given microorganism, calculated as prevalence in cases multiplied by the attributable fraction in the exposed [17]

**Supplementary table 2:** Microorganisms detected by PCR during the wet and dry seasons, from throat swabs in hospitalised children and matched controls, July-December 2016, using cutoff for detection Cq<40

|  | **Wet season^£^** | | | | | | **Dry season** | | | | | |
| --- | --- | --- | --- | --- | --- | --- | --- | --- | --- | --- | --- | --- |
| **Microorganism** | **Cases**  **N=184**  **n (%)** | **Controls**  **N=257**  **n (%)** | **Odds ratio (95%CI)** | ***P*-value*** | **AFE^#^,**  **% (95%CI)** | **AF^>^, %** | **Cases**  **N=123**  **n (%)** | **Controls**  **N=305**  **n (%)** | **Odds ratio (95%CI)** | ***P*-value*** | **AFE^#^,**  **% (95%CI)** | **AF^>^, %** |
| Viruses | | | | | | | | | | | | |
| Influenza A | 13 (7.1) | 1 (0.4) | 19.5 (2.5-150.1) | 0.004 | 94.9 (60.4-99.3) | 6.7 (4.3-7.0) | 12 (9.8) | 1 (0.3) | 32.9 (4.7-1409.1) | <0.001 | 96.9 (78.7-100) | 9.5 (7.7-9.8) |
| Influenza B | 8 (4.3) | 0 (0.0) | - | - | - | - | 6 (4.9) | 7 (2.3) | 2.2 (0.6-7.7) | 0.157 | 54.5 (0-87.0) | 2.7 (0-4.3) |
| Influenza A or B | 21 (11.4) | 1 (0.4) | 33.0 (4.4-247.6) | 0.001 | 97.0 (77.2-99.6) | 11.1 (8.8-11.4) | 18 (14.6) | 8 (2.6) | 6.4 (2.5-17.4) | <0.001 | 84.4 (60.0-94.3) | 12.3 (8.8-13.8) |
| RSV | 91 (49.5) | 11 (4.3) | 21.9 (11.2-42.7) | <0.001 | 95.4 (91.1-97.7) | 47.2 (45.0-48.3) | 9 (7.3) | 5 (1.6) | 4.7 (1.4-18.3) | 0.003 | 78.7 (28.6-94.5) | 5.7 (2.1-6.9) |
| HRV | 8 (4.3) | 5 (1.9) | 2.3 (0.7-7.1) | 0.152 | 56.3 (-35.6-86.0) | 2.4 (-1.5-3.7) | 9 (7.3) | 1 (0.3) | 24.0 (3.2-1055.1) | <0.001 | 95.8 (68.8-100) | 7.0 (5.0-7.3) |
| EV | 8 (4.3) | 3 (1.2) | 3.8 (1.0-14.7) | 0.049 | 74.0 (0.7-93.2) | 3.2 (0.0-4.1) | 7 (5.7) | 1 (0.3) | 18.3 (2.3-829.3) | <0.001 | 94.5 (56.5-99.9) | 5.4 (3.2-5.7) |
| HMPV | 2 (1.1) | 0 | - | - | - | - | 15 (12.2) | 0 | - | - | - | - |
| Bacteria | | | | | | | | | | | | |
| *H. influenzae* | 118 (64.1) | 80 (31.1) | 4.0 (2.6-5.9) | <0.001 | 74.7 (62.3-83.1) | 47.9 (39.9-53.3) | 77 (62.6) | 85 (27.9) | 4.3 (2.7-6.9) | <0.001 | 76.7 (63.0-85.5) | 48.0 (39.4-53.5) |

Seven participants had missing data for season

^£^wet season: May to October

**P*-value from logistic regression

**^#^**AFE: the proportion of cases found to be positive for a given microorganism, for whom the disease can be attributed to that microorganism, calculated as: 1 – (1/OR)

AF: the proportion of cases for whom the disease can be attributed to a given microorganism, calculated as prevalence in cases multiplied by the attributable fraction in the exposed [17]
